# Improved detection of *Mycobacterium tuberculosis* in lymph node aspirates through GeneXpert MTB/RIF assay in Bangladesh

**DOI:** 10.1186/s12879-026-12619-w

**Published:** 2026-01-22

**Authors:** Mohammad Sohel Shomik, Nishad Tasnim Mithila, Prakash Ghosh, M. Mamun Huda, Md. Rasel Uddin, Md. Golam Hasnain, Tanvir Ahmed Siddiqui, Syed Mohammad Mazidur Rahman, Kanu Lal Saha, Nasima Akhtar, Jason R. Andrews, Sayera Banu, Ahmed Abd El Wahed, Dinesh Mondal

**Affiliations:** 1 Nutrition Research Division, International Centre for Diarrhoeal Disease Research, Bangladesh, Dhaka 1212 Bangladesh; 2 Institute of Animal Hygiene and Veterinary Public Health, Leipzig University, Leipzig, Germany; 3 Institute for Social Science Research, University of Queensland, Brisbane, QLD Australia; 4 ARC Centre of Excellence for Children and Families Over the Life Course, University of Queensland, Brisbane, QLD Australia; 5 Faculty of Medicine, Hunter Medical Research Institute, University of Newcastle, Newcastle, Australia; 6 Department of Microbiology and Immunology, Bangladesh Medical University, Dhaka, Bangladesh; 7 Mycobacteriology Laboratory, Infectious Diseases Division, International Centre for Diarrhoeal Disease Research, Bangladesh (ICDDR,B), Dhaka, Bangladesh; 8 Department of Otolaryngology - Head and Neck Surgery, Bangladesh Medical University, Dhaka, Bangladesh; 9 Division of Infectious Diseases and Geographic Medicine, Stanford University School of Medicine, 300 Pasteur Drive, Stanford, CA 94305 USA; 10 School of Rural Medicine, Charles Sturt University, Orange, NSW Australia; 11 Department of Empirical Health Economics, Technische Universität Berlin, Berlin, Germany

**Keywords:** Tuberculous lymphadenitis, GeneXpert MTB/RIF assay, Diagnostic performance, Laboratory diagnosis

## Abstract

**Background:**

Despite tuberculous lymphadenitis (TBL) being the most prevalent type of extrapulmonary tuberculosis, there are limitations in laboratory diagnosis of TBL due to high cost, inadequate diagnostic efficacy and feasibility. Xpert MTB/RIF (Xpert) assay is a design-locked, molecular diagnostic technique that detects *Mycobacterium tuberculosis* (MTB) genome and rifampicin resistance by targeting *rpoB* gene and mutations within the gene. Currently, there is limited evidence validating Xpert assay usage for diagnosing TBL in Bangladesh. Therefore, in this study, we evaluated diagnostic efficacy of Xpert, considering composite reference standard (CRS), i.e. combination of acid-fast bacilli (AFB) microscopy, culture, Xpert assay and cytology, as gold standard, and compared it to cytology.

**Methods:**

AFB microscopy, culture, cytology, and Xpert assay were conducted on fine needle aspirates collected from 523 presumptive TBL patients. Genomic DNA was extracted from bacterial colonies of culture-positive specimens. In order to confirm presence of MTB, PCR and gel electrophoresis were performed on extracted DNA to detect RD9 region of MTB DNA. Sensitivity, specificity, positive and negative predictive values, and Cohen’s kappa coefficient were determined, and McNemar’s test was performed for Xpert and cytology with respect to CRS. Additionally, latent class analysis was performed to estimate sensitivity and specificity of all four diagnostic modalities.

**Results:**

Xpert showed sensitivity and specificity of 72.9% (261/358) and 100% (165/165) respectively against CRS, with 69.8% sensitivity and 97.1% specificity using Bayesian latent class modeling. In contrast, cytology demonstrated sensitivity and specificity of 84.1% (301/358) and 100% (165/165) against CRS, and 81.9% and 99.9% upon latent class analysis, respectively. Furthermore, Xpert showed moderate agreement with cytology (κ = 0.45, *p* < 0.0001), fair agreement with culture (κ = 0.30, *p* < 0.0001), and poor agreement with AFB microscopy (κ = 0.09, *p* < 0.0001).

**Conclusion:**

Our study findings validate routinely using Xpert assay in TBL diagnosis and enable detecting patients with low bacterial load. However, further assessment via cytology is recommended for confirmation in Xpert-negative patients having patent symptoms.

**Clinical trial:**

Not applicable.

## Introduction

Tuberculosis (TB) is an infectious bacterial disease occurring due to bacillus *Mycobacterium tuberculosis* (MTB), with more than 10 million TB infections occurring every year [1]. In general, MTB infects the lung causing pulmonary TB (PTB). However, the infectious bacterium often disseminates to extrapulmonary organs via the blood or lymph, resulting in extrapulmonary tuberculosis (EPTB) which accounts for 16% of total TB cases worldwide. Out of all forms of EPTB, tuberculous lymphadenitis (TBL) is most predominant, comprising 35–40% of EPTB cases globally [2, 3]. According to a clinical study in Bangladesh, TBL constituted 36.2% of EPTB cases [4].

In 2015, WHO incepted End TB Strategy with an overarching goal towards reducing TB mortality and incidence by 95% and 90% respectively by 2035. In accordance with WHO’s End TB Strategy, National Tuberculosis Control Programme (NTP) was established in Bangladesh with the aim of ensuring zero TB-associated morbidity and mortality. Despite continuous efforts, any preventive intervention or effective vaccine for TB is yet to be devised. Therefore, in order to curb TB transmission, NTP is dedicated to ensure early and accurate detection of infection followed by proper treatment management of TB cases. However, the Programme has been facing more challenges in early and confirmed diagnosis of EPTB than PTB due to variability in symptoms depending on affected organs [2, 5, 6]. Moreover, the paucibacillary nature of clinical samples obtained from infected inaccessible regions further complicates EPTB diagnosis [7].

For EPTB diagnosis, culture is the gold standard technique and is useful for detecting pathogen resistance to relevant drugs [5, 8]. However, results from culture take several weeks, delaying the diagnosis [9]. Conventionally, acid-fast bacilli (AFB) microscopy is used for detecting acid-fast mycobacteria but it has low sensitivity in the range of 0–40% and is unable to differentiate between MTB and nontuberculous *mycobacteria* (NTM) [10, 11]. In case of tuberculous lesions, fine needle aspiration cytology (FNAC) is initially performed for diagnostic purpose if the infected site is accessible. However, FNAC is unable to differentiate tuberculosis-associated lesions from other types of lesions [5, 10]. To overcome limitations of the aforementioned methods, a wealth of molecular assays, such as polymerase chain reaction (PCR) and real-time PCR have been maneuvered for amplifying and detecting MTB DNA. Although PCR techniques confer higher sensitivity than culture and AFB microscopy and have improved diagnostic accuracy, they require experienced personnel to operate and result in high number of false positives, limiting routine usage in EPTB diagnostics [5].

With the view to decentralize molecular assay and facilitate TB detection in low resource settings, a design-locked assay, Xpert MTB/RIF (Xpert) assay (Cepheid, USA) has been developed - a cartridge-based, automated molecular diagnostic technique that detects both MTB genome and mutations within it in 2 h [7, 12]. Based on principle of PCR, the assay targets the rifampicin resistance-determining region (RRDR) of *rpoB* gene in MTB. Amplification of this region helps in detecting presence of MTB or other mutated forms of the gene contributing to rifampicin resistance [12]. With the auspices of promising performance of the assay, in 2010 WHO first advocated using Xpert test for PTB diagnosis and in 2013, for EPTB diagnosis [13].

Initial diagnostic tests for TBL include AFB microscopy and cytology, with culture being the gold standard technique [14]. According to National Guideline and Operational Manual for Tuberculosis for Bangladesh, 2021, TBL diagnosis relies on Xpert assay, cultures, or AFB microscopy smears from lymph node aspirates [15]. Although a handful of research demonstrated diagnostic efficacy of Xpert testing TBL, limited data is available for the Bangladeshi population, warranting further diagnostic evaluation studies to discern efficiency of Xpert assay as routine technique in diagnosing TBL in Bangladesh. Thus, this study was performed for determination of diagnostic efficacy of Xpert assay in TBL patients of Bangladesh and comparing it with that of cytology.

## Methods

### Study sites and population

This cross-sectional study was conducted from 2013 to 2014 in collaboration with Ear, Nose, and Throat (ENT) Department and Microbiology Laboratory of Bangladesh Medical University, and Mycobacteriology Laboratory, of International Centre for Diarrhoeal Disease Research (icddr, b), Dhaka, Bangladesh. Approvals were obtained from Institutional Review Boards (IRBs) of both Bangladesh Medical University and icddr, b.

### Sample collection and transportation

The suspected TBL patients at Bangladesh Medical University were clinically assessed and informed consent was taken from them before enrolment into study. In addition, the TBL suspects treated with anti-TB drugs in the past four years were included in the study. Fine needle aspiration (FNA) was conducted twice per patient by trained pathologists under asceptic conditions. In each FNA procedure, the aspirate was collected in sterile 3 cc syringes where around 0.5–1 mL aspirate was collected from each patient. Following collection of aspirate, one portion was used for cytology, and another portion was used for Xpert, and the remaining portion was transported to icddr, b maintaining the cold temperature between 4 and 8 °C, and processed within 8 h of collection on the same day, for culture and AFB microscopy. In case of low volume of aspirate, phosphate-buffered saline (PBS) was used to rinse the aspirates trapped within the syringes, making the final volume up to 0.7–1 mL.

Malignant individuals identified by cytology were excluded from the study and immediately referred to respective Bangladesh Medical University department for appropriate management.

### Cytology

0.1 mL of collected aspirates was used for cytology. Cytology was performed following methodology in previous literature [16, 17]. The FNA smears were air dried for 15 min, and then stained with the hematoxylin solution for 4 min. Tap water was used to rinse the stained slides for removing any unbound hematoxylin. Differentiation was done twice with acid alcohol (10% acetic acid and 85% ethanol in water), and then the slides were again rinsed with tap water. Lithium carbonate solution was used to saturate the tissues and rinsed with tap water. Finally, after counterstaining the slide with eosin azur for 2 min, it was rinsed twice, once with 95% alcohol and second time with 100% alcohol-diluted. The slides were examined by two pathologists and in case of any discrepancy between results, the confusion was resolved by a third pathologist.

### Xpert MTB/RIF assay

A trained laboratory officer at Bangladesh Medical University performed the Xpert assay according to the methodology defined in previous literature [23]. Through this assay, *rpoB* gene of MTBC and rifampicin resistance were detected. According to the procedure, 0.7 mL of PBS-diluted specimen was mixed in 1:2 ratio with sample reagent buffer of Xpert MTB/RIF (Cepheid, Sunnyvale, CA, USA) assay in a 15 mL microcentrifuge tube, vortexed and then incubated for 15 min at room temperature with regular gentle vortexes. After that, 2 mL of incubated suspension was taken in Xpert cartridge and eventually placed in the Xpert machine. Following completion of the assay, results generated by the Xpert software were recorded.

### Specimen processing for AFB microscopy and culture

To perform microscopy and culture, the specimens were processed in accordance to method described previously [18]. Approximately 0.1 mL of PBS-diluted lymph node aspirate samples were sent from Bangladesh Medical University to ICDDR, B Mycobacteriology Laboratory for AFB microscopy and culture. The samples were treated via N-acetyl-L-cysteine– sodium hydroxide (NALC - NaOH) method which involved addition of equivalent volumes of 1.45% sodium citrate and 2% NaOH along with 0.5% NALC to specimens for simultaneous digestion and decontamination of samples. Resulting mixtures underwent vortexing and incubation for 15 min at room temperature, with regular shaking by hand. Following incubation, the decontaminated samples were diluted with PBS of pH 6.8 and centrifuged for 15 min at 3000xg. Following removal of supernatant from the centrifuged samples, 1.5 mL PBS was added for re-suspending the pellet formed at the bottom. The final suspensions were then used for smear preparation and for inoculation into Lowenstein-Jensen (L-J) media slant for culture.

### AFB microscopy

Smears for AFB microscopy were prepared using the concentrated processed specimens as indicated by Uddin et al. [19]. Processed sputum was taken using a 10 mm inoculation loop, and smeared on slide over an area of approximately 2.0 by 1.0 cm. The smear underwent air-drying, heat fixation, and staining with Ziehl-Neelsen (ZN) stain, and was observed under light microscope (at 1000x magnification). Samples were regarded as AFB-negative if no bacilli were found in at least 100 microscopic fields and AFB-positive if at least 1–9 bacilli were found in 100 microscopic fields.

### Culture and drug susceptibility testing

Both culture and drug susceptibility testing (DST) were done according to methods described by Uddin et al. and Ghariani et al. [18, 20]. Two loops (20 µL) of processed specimens were inoculated into two L-J slants and incubated vertically at 37 °C for 8 weeks. Sample was regarded culture-positive upon visibility of rough, buff-colored colonies against the green media and culture-negative upon observation of no colony within 8 weeks’ time.

For DST, standard proportion method was used against four anti-TB drugs with the following concentrations: 4 mg/L of streptomycin, 40 mg/L of rifampicin, 0.2 mg/L of isoniazid, and 2 mg/L of ethambutol. When the growth rate of an isolate was observed to be less than 1% in drug-containing media compared to the drug-free control, it was considered sensitive. Similarly, growth rate of 1% or greater compared to the control indicated that the isolate was resistant to a specific drug.

### Genomic DNA extraction, PCR and gel electrophoresis

Genomic DNA isolation from *Mycobacterium* isolates was done in accordance to method as per Uddin et al. [18]. Bacterial colonies from fresh cultures of only culture-positive specimens were suspended in 100–200 µL distilled water and dry heating continued for 30 minutes at 95°C. Centrifugation was done for 5 minutes at 10,000 rpm and supernatant containing genomic DNA were obtained and stored at -20°C. In order to confirm presence of MTB in culture-positive specimens, PCR was performed to amplify the Region of difference 9 (RD9) region (333 bp) using the forward primer (5’-CGATGGTCAACACCACTACG-3’) and reverse primer (5’-CTGGACCTCGATGACCACTC-3’), based on methodology by Brosch et al.,2002 [21]. For every PCR run, positive control (DNA from MTB H37Rv strain) and negative control (nuclease-free water) were utilized. Amplicons generated from PCR were separated and identified using 1.5% agarose gel electrophoresis, stained with ethidium bromide, and imaged via Gel Doc XR system (Bio-Rad) [22].

### Case definitions

The cases of TBL were regarded as suspected TBL patients testing positive by at least one of the four laboratory diagnostics (culture, AFB microscopy, cytology, and/or Xpert assay) used in the study.

On the contrary, controls were defined by suspected TBL individuals testing negative by all four laboratory techniques.

True positive cases by CRS were defined as enrolled TBL suspects testing positive by Xpert assay, cytology, culture, and/or AFB microscopy.

In contrast, true negatives by CRS were characterized by TBL suspects testing negative via the aforementioned four diagnostic methods.

### Statistical analysis

Continuous variables were summarized as means and standard deviations whereas categorical data were represented with absolute numbers as well as percentages. Cohen’s kappa coefficient values were calculated and McNemar’s tests were performed for observing agreement and disagreement between two diagnostic modalities respectively. Diagnostic efficacy parameters of Xpert assay and cytology were determined considering CRS as gold standard. All statistical analyses were performed in RStudio (version 2023.12.1 + 402) with p-value < 0.05 considered statistically significant.

### Latent class analysis

Sensitivity and specificity of each diagnostic technique were estimated using Bayesian latent class models. The following prior estimates of sensitivity (95% CIs) were used for the tests − 23–40% for AFB microscopy [23–25], 30–60% for culture [26, 27], and 64-88.5% for cytology [23] and uninformative sensitivity for Xpert assay. As for specificity, priors (95% CIs) of 98–100% were considered for culture, AFB microscopy, and Xpert [24, 28], and 50.7–66.5% for cytology [23]. The data from the study had multinomial distribution and sampling was done with 100,000 iterations using Markov Chain Monte Carlo (MCMC) approach, removing top 50,000 observations and utilizing the remainder for inferring the diagnostic efficacy of the tests. The results were reported in median along with 95% credible intervals.

## Results

### Study participants’ characteristics

**Fig. 1:**
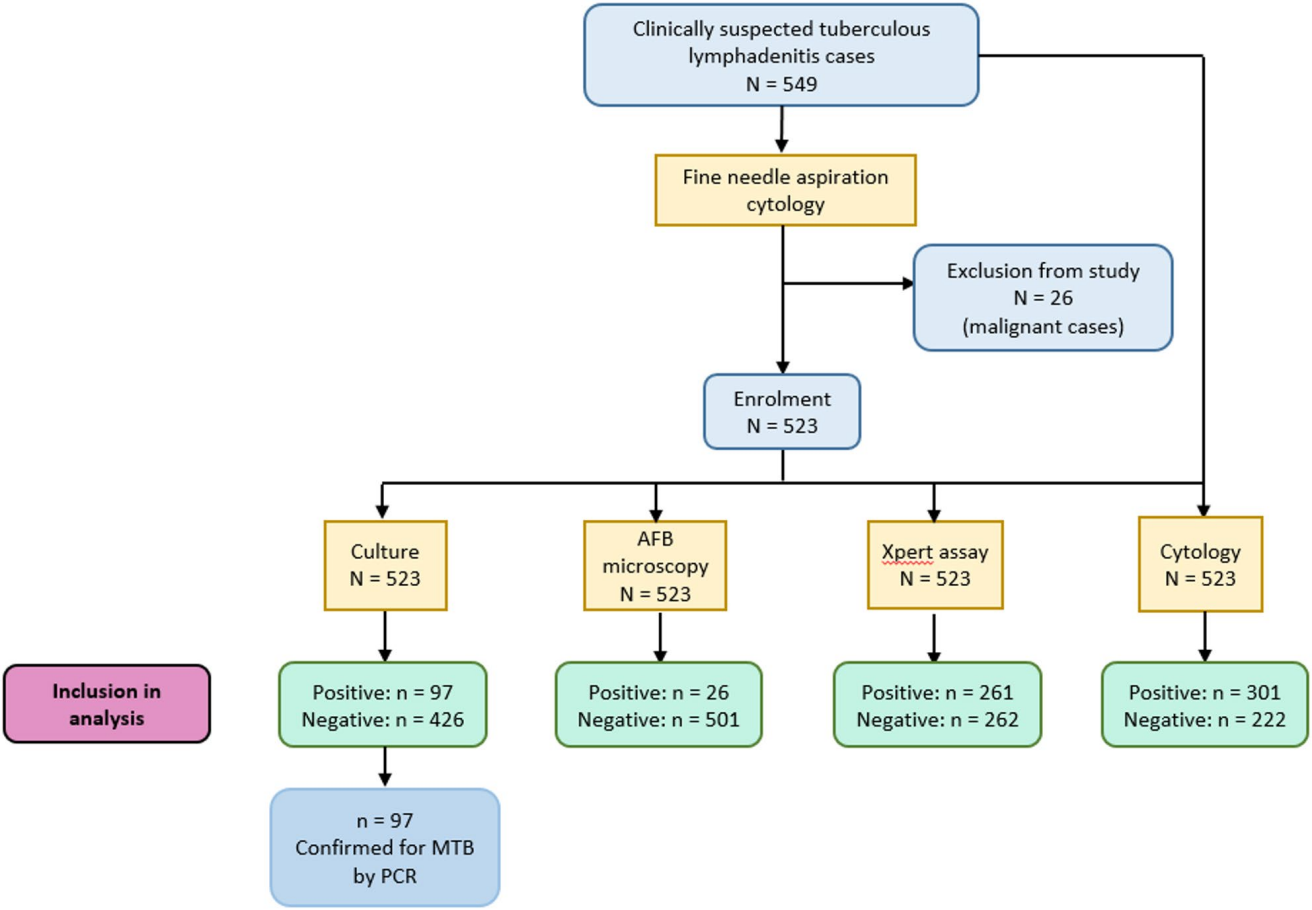
Study flowchart depicting enrolment and subsequent laboratory diagnosis of study participants

549 patients suspected for TBL were recruited in the study; 26 malignant cases were identified by FNAC and ultimately excluded from the study. Finally, laboratory diagnosis for detection of MTB was performed on the remaining 523 non-malignant patients and their clinical metadata was subjected to analysis (Fig. 1).

According to our study, there was an overall female preponderance with percentages of females and males being 60.6% and 39.4% respectively. Mean age was 27.98 years of all 523 suspected TBL patients, with majority aged 18–45 years followed by children of age below 18 years. Ratio of patients suffering from high-grade (greater than 102^o^F) to that with low-grade fever (equal to or less than 101^o^F) was 11:4. Among the enrolled cases, 80 (15.3%) cases had past history of tuberculosis (Table – 1).

**Table 1:** Demographic and clinical parameters of all enrolled study participants with and without TBL

Indicators	Case (*N* = 358)	Control (*N* = 165)	*p* value	Total (*N* = 523)
Age (in years), Median(Interquartile range)	26.5 (21–35)	25 (15–33)	0.22	N/A
Body Mass Index (BMI) [in kg/m^2^], Median(IQR)	20.9 (18.3–23.7)	20.8 (18.2–23.9)	0.71	N/A
Temperature (in ^o^F), Median (IQR)	98.0 (98.0–99.0)	98.0 (98.0–99.0)	0.85	N/A
Gender, % (n) - Female - Male	61.5 (220) 38.5 (138)	58.8 (97) 41.2 (68)	0.63	60.6 (317) 39.4 (206)
Fever, % (n) - Present	36.3 (130)	41.8 (69)	0.27	38.0 (199)
BCG mark,% (n) - Present	50 (179)	49.7 (82)	1	49.9 (261)
Pallor, % (n) - Present	70.7 (253)	72.7 (120)	0.70	71.3 (373)
Median Size of lymph nodes (in cm), Median(IQR)	2(1–3)	1(0.5–2)	**0.005**	N/A
Tenderness of lymph nodes, % (n) - Present	39.1 (140)	40(66)	0.92	39.4 (206)
Mobility of lymph nodes, % (n)	77.1(276)	80.6 (133)	0.43	78.2 (409)
Past history of tuberculosis, % (n)	17.3(62)	10.9 (18)	0.08	15.30 (80)
Smoking, % (n)	10.3 (37)	7.9 (13)	0.47	9.56 (50)
Alcohol intake, % (n)	0.8 (3)	0.6 (1)	1	0.76 (4)

The patient data collected in our study demonstrated that palpable lymph nodes of the patients were distributed across different body sites, occurring most frequently in submandibular space (29.3%) and the least in superior mediastinum (0.2%). In 5.2% of the cases, the palpable lymph nodes were present in multiple sites. The mean size of lymph node was 1.73 ± 1.29 cm and subsequently stratified based on their consistencies. Approximately 70% of patients involved firm lymph nodes constituting majority of the cases, followed by 20.8% with hard lymph nodes and 8.6% with soft lymph nodes. Majority of the patients with lymph node TB were underweight and mildly anemic whereas severe anemia was reported in only one patient.

Out of the 523 enrolled cases, 358 (68.5%) cases were positive for *Mycobacterium tuberculosis* via at least one of the diagnostic methods including AFB microscopy, cytology, culture, and Xpert assay. Within 358 TB-positive cases, there were 26 (7.3%) positive cases were detected by AFB microscopy, 97 (27.1%) by culture, 301 (84.1%) by cytology, and 261 (72.9%) by Xpert test.

### Diagnostic efficacy of Xpert assay

Upon evaluation against composite reference standard (CRS), sensitivity, specificity, positive predictive value (PPV), negative predictive value (NPV), and diagnostic accuracy of Xpert assay were 72.9%, 100%, 100%, 63.0%, and 81.5% respectively (Table – 2). Xpert assay showed significantly substantial agreement with CRS.

**Table 2:** Diagnostic efficacy of all four diagnostic methods in the study - Xpert MTB/RIF assay and cytology with respect to composite reference standard

		Composite reference standard		Sensitivity (95% CI)	Specificity (95% CI)	Positive predictive value (95% CI)	Negative predictive value (95% CI)	Accuracy (95% CI)	Kappa coefficient	p-value in agreement analysis	p-value in McNemar test
		Positive	Negative								
Xpert MTB/RIF assay	Positive	261	0	72.9 (68.0–77.4)	100 (97.8–100)	100 (98.6–100)	63.0 (56.8–68.8)	81.5 (77.9–84.7)	0.63	< 0.0001	< 0.0001
	Negative	97	165								
Cytology	Positive	301	0	84.1 (79.9–87.7)	100 (97.8–100)	100 (98.8–100)	74.3 (68.1–79.9)	89.1 (86.1–91.6)	0.77	< 0.0001	< 0.0001
	Negative	57	165								

Moreover, Xpert presented different degrees of concordance with reference diagnostic modalities constituting the CRS. Among 261 Xpert-positive cases, 209 (80.1%) were cytology-positive. This was followed by 87 cases (33.3%) which were positive by culture. In total, there were 26 microscopy-positive cases out of which 25 (9.6%) were positive via Xpert test. On the other hand, Xpert assay displayed kappa coefficients of 0.45(*p* < 0.0001) with cytology, 0.30(*p* < 0.0001) with culture, and 0.09(*p* < 0.0001) with microscopy.

### Diagnostic performance of cytology

Cytology detected the TBL cases with sensitivity as high as 84.1% (95% CI: 79.9–87.7) and identified true negative cases with a specificity of 100% (95% CI: 97.8–100). According to our study, the PPV and NPV of cytology were 100% and 74.3% respectively. Cytology also exhibited significantly substantial agreement with CRS (Table – 2).

### Latent class modeling

Using Bayesian latent class modeling, Xpert assay showed an estimated sensitivity of 69.8% (95% credible intervals [CrI]: 64.7–74.6) and specificity estimate of 97.1% (95% CrI: 89.7–99.9). Low sensitivity of 26.5% were reported for culture. An elevated sensitivity of 81.9% was estimated for cytology according to the latent class analysis whereas sensitivity of AFB microscopy was estimated to be the poorest out of the four diagnostic modalities at 7.2 (95% CrI: 4.9–10.2). Culture, AFB microscopy and cytology exhibited a common specificity of 99.9% (Table − 3).

**Table 3:** Median sensitivities and specificities with 95% credible intervals (95% CrI) of all four laboratory diagnostics estimated using Bayesian latent class modeling

Diagnostic modality	Sensitivity, % (95% CrI)	Specificity, % (95% CrI)
Xpert MTB/RIF assay	69.8 (64.7–74.6)	97.1 (89.7–99.9)
Culture	26.5 (22.1–31.3)	99.9 (99.6–99.9)
Acid-fast bacilli microscopy	7.2 (4.9–10.2)	99.9 (99.7–99.9)
Cytology	81.9 (76.8–86.7)	99.9 (99.6–99.9)

### Drug resistance profile of TBL suspects

A total of 24 drug-resistant cases were identified via both phenotypic DST and Xpert assay. Out of 17 drug-resistant cases detected by DST, 2 cases showed resistance to all four DST drugs while 2 other cases showed resistance to streptomycin, isoniazid, and rifampicin. Furthermore, only 2 cases were streptomycin- and isoniazid-resistant and only one of them were resistant to both isoniazid and ethambutol. In contrast, 10 cases were resistant to streptomycin only. On the other hand, Xpert assay detected 9 rifampicin-resistant cases in total. Interestingly, Xpert assay was able to detect 2 out of 4 rifampicin-resistant cases identified by DST. No bacterial growth was observed via L-J culture in 7 Xpert-positve rifampicin resistant cases and therefore, DST could not be performed (Table − 4).

**Table 4:** Drug resistance profile of the enrolled TBL suspects determined via Xpert assay and DST. The letter “**R**” represents resistant, “S” represents susceptibility to respective anti-TB drugs, “*ND*” represents drug resistance/susceptibility status not determined

Patient ID	Rifampicin Resistance by Xpert assay	Drug resistance by DST			
		Streptomycin	Isoniazid	Rifampicin	Ethambutol
1	S	**R**	**R**	**R**	S
2	**R**	**R**	**R**	**R**	S
3	S	**R**	**R**	S	S
4	S	**R**	**R**	S	S
5	S	**R**	S	S	S
6	S	**R**	S	S	S
7	S	**R**	S	S	S
8	S	**R**	S	S	S
9	S	**R**	S	S	S
10	S	**R**	S	S	S
11	S	**R**	S	S	S
12	S	**R**	S	S	S
13	S	**R**	S	S	S
14	S	**R**	S	S	S
15	S	S	**R**	S	**R**
16	S	**R**	**R**	**R**	**R**
17	**R**	**R**	**R**	**R**	**R**
18	**R**	*ND*	*ND*	*ND*	*ND*
19	**R**	*ND*	*ND*	*ND*	*ND*
20	**R**	*ND*	*ND*	*ND*	*ND*
21	**R**	*ND*	*ND*	*ND*	*ND*
22	**R**	*ND*	*ND*	*ND*	*ND*
23	**R**	*ND*	*ND*	*ND*	*ND*
24	**R**	*ND*	*ND*	*ND*	*ND*

## Discussion

Extrapulmonary tuberculosis (EPTB) has been known to constitute 15–19% of all TB cases in Bangladesh, with lymph node being the most frequently affected region [29, 30]. However, clinical diagnosis of TBL is quite challenging as disease manifestations resemble those of other pathologies, such as fungal infections and inflammations [31]. In such cases, existing laboratory diagnostics serve as useful alternatives but they have a few shortcomings limiting their routine usage. In 2013, WHO certified Xpert assay for EPTB diagnosis [32]. Until now, numerous studies have been performed for determining diagnostic performance of Xpert assay involving EPTB specimens in different parts of the world, including Bangladesh [31]. However, there is inadequate evidence on diagnosing TBL patients in Bangladesh using this particular assay. The overarching goal of this study was to explore diagnostic efficacy of Xpert assay in the context of TBL in comparison to cytology with respect to CRS in a low- and middle-income country like Bangladesh.

In our study, similar sensitivity and specificity values of Xpert assay were obtained against CRS and using Bayesian latent class model in absence of gold standard. This noted sensitivity is similar to those found in multiple studies involving lymph node aspirates as specimen type for detecting MTB and culture or CRS being considered the gold standard [27, 33–38]. Our study results indicate an improved sensitivity of Xpert test for diagnosing TBL in Bangladesh compared to other countries in the Indian Subcontinent. This improved sensitivity may be attributed to the selection of a less sensitive L-J culture, uneven distribution of lymph node aspirates, and sample processing, which, in turn, might have influenced the true diagnostic performance of culture and AFB microscopy. Alternatively, the high specificities for the assay fell within the range reported in high TB-burden countries including India, China, Tanzania, Ethiopia, and South Africa [33, 36, 38–44].

Xpert assay was able to detect MTB DNA in 174 culture-negative specimens. Difference between Xpert and culture results could be explained by use of less sensitive L-J culture media. Furthermore, the disccordance could be due to Xpert’s potential to detect *rpoB* gene fragment in MTB from both viable and dead bacilli in contrast to culture, which allows growth of only viable bacilli. The reasoning can be further supported by the data on anti-TB treated study participants (*n* = 66) who tested positive more via Xpert (*n* = 41) than culture (*n* = 5). It is also possible that viable bacilli in the samples were lost during sample processing prior to culture, resulting in poor bacilli quantity in the representative specimens.

Xpert assay had the highest agreement with cytology (κ = 0.45, *p* < 0.0001), among three other laboratory diagnostics including cytology, culture, and microscopy. Similar moderate agreement were observed between Xpert and cytology ranging 0.40–0.60 [45, 46].

In comparison to Xpert assay, cytology demonstrated higher sensitivity against the CRS and using Bayesian latent class modeling. As cytology mainly focuses on cytomorphological changes rather than bacteriological detection, the technique cannot differentiate between tuberculous and nontuberculous granulomas (e.g. inflammatory granulomas), therefore leading to high number of cytology-positive cases in the study.

Xpert assay and cytology exhibited excellent specificities and PPVs of more than 97% against CRS and using latent class analysis. The patent symptoms of TBL in patients prior to testing indicated high pre-test probability among the enrolled suspected patients, eventually resulting in tests showing high specificities and PPVs in the study.

Xpert assay was able to detect more rifampicin-resistant cases than DST. This can be explained by inadequate amount of aspirate used for culture in case of Xpert-positive samples as Xpert assay was prioritized over other diagnostic methods. As previously mentioned, Xpert assay accounts for presence of both dead and viable bacilli whereas DST involves only viable bacilli [47, 48]. This particular nature of assays might have been responsible for Xpert assay’s successful detection of low bacterial load and identification of rifampicin resistance in both dead and viable bacilli unlike DST [49]. Despite higher detection of resistance, Xpert assay failed to detect 2 out of 4 DST-identified rifampicin resistant cases. The underlying reason for this discrepancy might be due to presence of mutations outside the RRDR region of *rpoB* gene, responsible for conferring rifampicin resistance during DST.

Overall, the Xpert and DST results from our study indicated persistence of drug-resistant TBL (DR-TBL) in Bangladesh which has also been reported in previous studies from different regions worldwide [50–52]. Drug-resistant EPTB (DR-EPTB) is often overlooked and can lead to serious health consequences if untreated [2, 29, 53, 54]. Similarly, DR-TBL can result in poor treatment outcomes, resulting in treatment failure and/or adverse drug effects [52, 53]. Simultaneously, the infected lymph nodes can serve as sites for mycobacterial growth and disseminate bacteria to other organs, leading to other forms of EPTB [55]. This necessitates ensuring early and confirmed DR-TBL diagnosis for early and proper administration of sensitive anti-TB drugs to patients and consequent management of the disease. As Xpert assay detects rifampicin resistance with higher sensitivity and shorter time than DST, it can play a significant role in DR-TBL diagnosis.

All the concerned physicians were informed of the laboratory test results of study participants in written form. The concerned physicians reported the laboratory test results to respective study participants. Out of 523 study participants, all 358 TB patients testing positive by at least one of the four laboratory diagnostics were referred to the Internationally recommended strategy for TB control centers (DOTS centers) for treatment according to the protocol by NTP [56].

In our study, ratio of female to male study participants is 1.6:1 which is concordant with some of the previous studies involving TBL [23, 25, 57]. Although the exact reasons underlying this phenomenon remains unelucidated to date, some factors are presumed to be particularly associated with enhanced risk to females. They include weak immune system, hormone-specific effects, low nutritional intake, high genetic susceptibility of organs to the disease, practices that facilitate exposure to MTB (e.g. milking infected cows) and limited access to healthcare [58, 59]. The age of majority of the patients were between 18 and 45 years. This closely agreed with age ranges of 20–30 and 30–40 years of patients with TBL [57–61].

The unique attributes of this study were inclusion of large sample size of suspected TBL patients and use of CRS and Bayesian latent class analysis for estimating sensitivity and specificity of all four different diagnostic modalities in the study. Although CRS combines results from multiple tests and is commonly used by researchers for classifying diseased and non-diseased individuals, a drawback involving CRS is the assumption of similar accuracy of component tests. This is due to failure in considering unique accuracies of the component tests and can lead to biasness in accuracy estimates. Therefore, to overcome this issue, Bayesian latent class modeling was also applied to the study data, which took into account prior estimates of accuracy of each component test [62].

Likewise, our study also had a few limitations. Our study yielded compromised efficiencies for L-J culture and AFB microscopy. Since Xpert assay was the index method in this study, it was prioritized for aspirate use over other diagnostic modalities. As a result of inequal sample distribution, inadequate volumes of aspirate were available for performing culture and microscopy. Additionally, sample processing resulted in variable bacterial loads for the same sample, resulting in biased and reduced diagnostic efficiencies of both the techniques. Eventually, although we observed comparable diagnostic efficacy of Xpert with previous studies, it was not the case for AFB microscopy and culture in some instances. Therefore, CRS was chosen to be the gold standard instead of L-J culture for determining diagnostic efficacies of Xpert assay and cytology in our study. The impact of sample volume on diagnostic outcomes have been shown in prior studies where increase in sample volume led to increased culture positivity and recovery rate of bacteria in culture [63, 64]. Similarly, a study involving AFB microscopy showed significant increase in smear positivity with increase in sputum volume [65]. On the other hand, different consistencies of aspirates were collected which might have affected the diagnostic outcomes of the study. This is supported by observations from previous studies - presence of blood during sample collection diluted aspirates, resulting in inadequate aspirate volume [66, 67]. Compared to hemorrhagic aspirates, caseous and purulent lesions found at later stage of TB infection contain more bacilli and show higher MTB detection rates via Xpert assay [14, 38]. Moreover, we were unable to follow up the TBL patients in the study for ensuring definitive treatment and cure. Inclusion of data on positive response to anti-TB treatment in patients could help in confirmation of true positive cases and therefore, provide a more accurate estimate of diagnostic performance of all diagnostic modalities including Xpert assay. Therefore, further longitudinal study is required for addressing this particular gap. Another limitation is the inclusion of TB-treated individuals in the study. Laboratory data and analyses from only treatment-naïve study participants would provide stronger evidence of Xpert assay detecting true-positive TB cases that were, otherwise, missed by culture. Furthermore, recently Xpert MTB/RIF Ultra (Ultra) assay has been developed with increased sensitivity, having a limit of detection (LOD) of 15.6 colony-forming units(CFU)/mL in comparison to Xpert assay with LOD of 131 CFU/mL [68]. In 2017, WHO recommended replacement of Xpert cartridges with Ultra due to higher sensitivity in detecting patients with very low bacterial load [69]. However, future studies are required to validate use of Ultra assay for TBL diagnosis.

It is important to note that the discrepancy in results involving Xpert assay, culture and smear microscopy could be reduced by using liquid *Mycobacteria* Growth Indicator Tube (MGIT) culture media and fluorescent microscopy which are more sensitive than techniques used in the study, L-J culture media and ZN smear microscopy respectively. Furthermore, our study could be refined methodologically by sequencing the *rpoB* gene region of MTB DNA, confirming drug-resistant cases and identifying mutations in *rpoB* gene responsible for rifampicin resistance.

Existing methods for diagnosing TBL are time-consuming, expensive and are of low diagnostic performance, further requiring complex laboratory setup. Although culture is the gold standard for TB diagnosis, Xpert provides results for both TB detection and rifampicin resistance within approximately 90 min, offering a substantial advantage over L-J culture, which takes several weeks to yield results [39]. Xpert assay is more feasible for usage in low-resource settings like Bangladesh and has previously been used as districtwide diagnostic platform for detecting PTB cases. Currently, TBL is diagnosed via Xpert assay on FNAC samples in accordance to the National Guideline and Operational Manual for Tuberculosis [15]. The present findings of study validate use of Xpert testing diagnosing TBL patients, showing significant congruence with CRS. Xpert can detect patients with low bacterial load that would otherwise be undetected by conventional diagnostics, consequently receiving timely appropriate treatment and further preventing disease progression and transmission in the community.

## Conclusion

Our study findings validate routine use of Xpert assay for TBL diagnosis in Bangladesh. However, Xpert assay must not be considered a standalone test for diagnosing TBL. In case of Xpert-negative patients with patent symptoms, further assessment via cytology is recommended for confirmation in the reference facility.

## Data Availability

The research data supporting the results of this study can be obtained following reasonable request to the corresponding author.

## Abbreviations

AFB: Acid-fast bacilli
CFU: Colony-forming units
CRF: Case Report Form
CRS: Composite reference standard
DNA: Deoxyribonucleic acid
DR-EPTB: Drug-resistant extrapulmonary tuberculosis
DR-TBL: Drug-resistant tuberculous lymphadenitis
DST: Drug susceptibility test
EPTB: Extrapulmonary tuberculosis
FNA: Fine needle aspiration
FNAC: Fine needle aspiration cytology
icddr,b: International Centre for Diarrhoeal Disease Research, Dhaka
IRB: Institutional Review Boards
LiPA: Line probe assay
L-J: Lowenstein-Jensen
LOD: Limit of detection
MCMC: Markov Chain Monte Carlo
MTB: Mycobacterium tuberculosis
NALC-NaOH: N-acetyl-L-cysteine-sodium hydroxide
NPV: Negative predictive value
NTP: National Tuberculosis Control Programme
PBS: Phosphate-buffered saline
PCR: Polymerase chain reaction
PPV: Positive predictive value
PTB: Pulmonary tuberculosis
TB: Tuberculosis
TBL: Tuberculous lymphadenitis
TB-LAMP: Loopamp™ MTBC Detection Kit
WHO: World Health Organization
ZN: Ziehl-Neelsen
